# Age-specific sex effects in extinction of conditioned fear in rodents

**DOI:** 10.3389/fnbeh.2023.1298164

**Published:** 2023-12-13

**Authors:** Sajida Malik, Chun Hui J. Park, Jee Hyun Kim

**Affiliations:** ^1^School of Medicine, Institute for Mental and Physical Health and Clinical Translation, Deakin University, Geelong, VIC, Australia; ^2^Florey Department of Neuroscience and Mental Health, The University of Melbourne, Parkville, VIC, Australia

**Keywords:** associative learning, extinction, sex, development, maturation, aging, conditioned fear, memory

## Introduction

A hallmark of anxiety-related disorders is difficulties in inhibiting irrational and excessive fear. Fear and anxiety usually occur as a result of internal or external cues (Anderson and Adolphs, 2014). For example, panic disorder involves sudden and internal feeling of impending doom and pounding heart, while arachnophobia involves encountering spiders or spider-like objects in the environment. However, repeated exposure to fear-eliciting cue without any threatening results can lead to reduced fear to the cue, referred to as fear extinction (Maren et al., 2013; Ganella and Kim, 2014). Extinction is the process underlying exposure therapies to treat anxiety disorders, and it is observed across species, including in humans and in rodents (Kim and Ganella, 2015). Conservation of fear extinction across species has allowed numerous discoveries in the past few decades to promote our understanding of the neurobiology of anxiety-related disorders and their treatment (Maren et al., 2013; Haaker et al., 2019).

**Figure 1:**
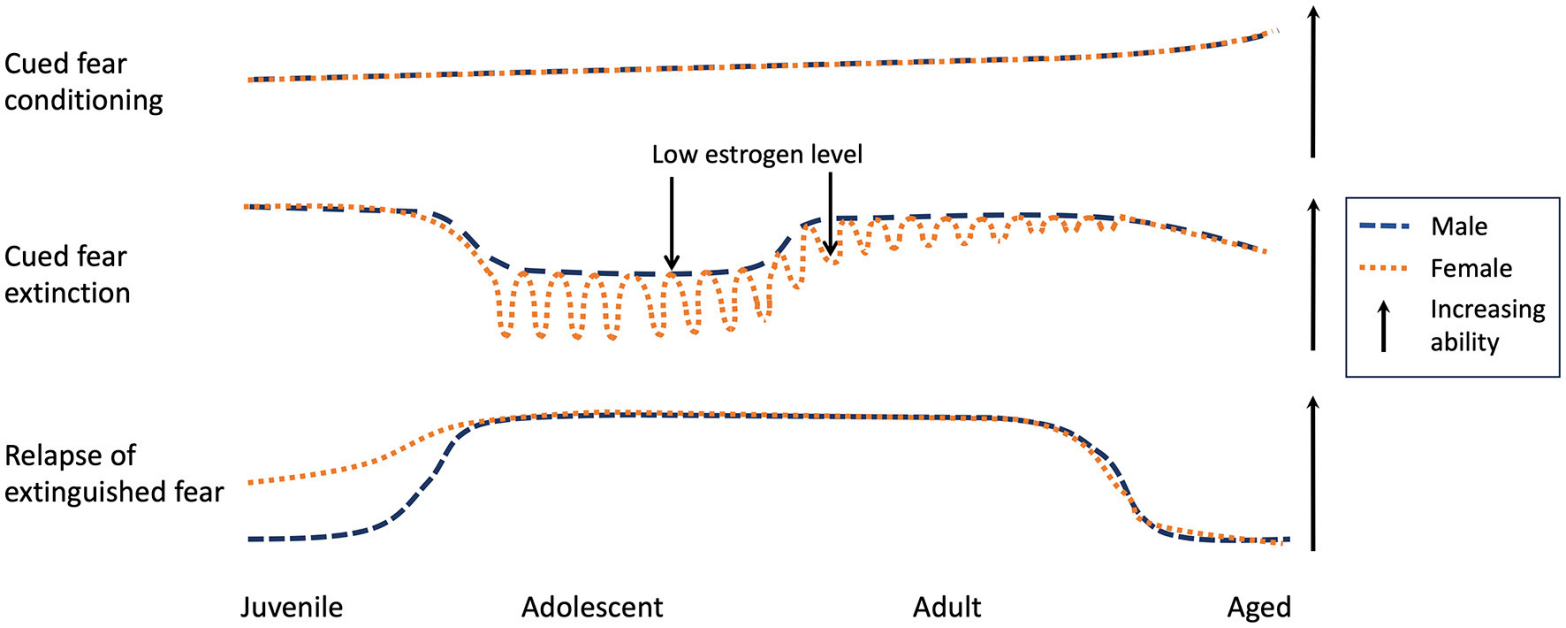
Ability in cued fear conditioning, extinction, and relapse of extinguished fear across the lifespan in male and female rodents (Park et al., 2017a,b, 2020a,b; Perry et al., 2020; Drummond et al., 2021; Short et al., 2022). Findings are generally consistent that there are no sex differences in cued fear conditioning throughout life in rodents. For extinction, there are distinct sex differences between males and females across life, with evidence of estrogen playing opposing roles in adolescence compared to adulthood. Relapse of extinguished fear is robust in juvenile females but absent in males. Interestingly, middle-aged rodents do not show reinstatement, suggesting they may revert to juvenile-like behaviors.

One significant impediment against translation of preclinical findings to clinical research has been the historical focus on male rodents in neurobiological research into fear extinction. It is speculated that the male focus has been to avoid potential variability in data due to the cycling hormones in female rodents, with neuroscience showing the biggest bias (5.5 male to 1 female studies) in biological disciplines (Zucker and Beery, 2010). Consequently, existing preclinical research fails to capture the demographic of anxiety disorders that are diagnosed twice more in females than males (Kessler et al., 2005; Zucker and Beery, 2010). To address this issue, recent studies have examined fear learning and extinction in adult female rodents. For example, extinction in adult female rats is facilitated during proestrus phase associated with high levels of estrogen (Milad et al., 2009), with systemic injection of estradiol following extinction leading to facilitated extinction recall (Zeidan et al., 2011). These reports of estrogen enhancing extinction is counter-intuitive considering that females have ~5 times more estrogen than males (Frederiksen et al., 2020) but there is a higher prevalence of anxiety disorders in females compared to males (McLean et al., 2011). However, sex-specific findings in adult rodents do not address the human epidemiology adequately. Anxiety-related disorders are typically developmental in their origin (Mineka and Zinbarg, 2006) and onset of anxiety disorders is the most prevalent during childhood and adolescence (Kessler et al., 2007). Notably, high prevalence of anxiety disorders in females over males is observed as young as 6 years of age (Lewinsohn et al., 1998) and such sex difference persists into aging (Vasiliadis et al., 2020). Therefore, we seek to provide an overview of sex-specific rodent research in fear conditioning and extinction processes during the juvenile period, adolescence, and in aging (see summary of published findings in Figure 1). Male and female descriptions refer to rodent studies and any reference to human studies were explicitly stated in this opinion article. Studies that directly compare or concurrently examine males and females to report them separately will be prioritized, highlighting the critical importance of age-specific sex difference research in translational psychiatry.

## Extinction as a new inhibitory learning that competes with the fear memory

Fear learning or conditioning typically involves the presentation of a neutral cue (e.g., tone) paired with a threatening stimulus (e.g., electric shock) (Maren et al., 2013; Ganella and Kim, 2014). This is repeated till the conditioned cue alone induces fear responses, such as “freezing”, an absence of movement other than respiration in rodents (Blanchard and Blanchard, 1969). Fear responding to the cue is taken as evidence of fear memory associated with the cue (Maren et al., 2013; Ganella and Kim, 2014). Fear extinction involves exposure to such a cue without any threatening outcomes, which attenuates the cue-induced fear response (Maren et al., 2013; Ganella and Kim, 2014). Extinction is generally a new memory that interferes with the expression of the fear memory (Maren et al., 2013; Ganella and Kim, 2014). In support of this idea are three widely observed phenomena when conditioned fear after extinction can relapse (Maren et al., 2013; Ganella and Kim, 2014). Renewal refers to when fear returns upon conditioned cue exposure in a different context from extinction. Reinstatement refers to when exposure to a reminder, such as a mild threatening stimulus, causes fear expression to the extinguished cue. Spontaneous recovery refers to when the conditioned fear response is expressed due to the passage of time since extinction.

## Juvenile sex differences in extinction of conditioned fear

Cognitively, juvenile period in rodents represent young childhood in humans before large scale abilities (e.g., physically navigating a maze rather than a computer screen-based spatial task) fully emerge (Overman et al., 1996; Madsen et al., 2016). During this time, there is evidence of stronger contextual conditioned fear in juvenile males compared to females (Park et al., 2017b, 2020a). In contrast, cued fear acquisition did not differ between postnatal day (P) 18 male and female rats (Park et al., 2017a). In that study, fear retrieval and the rate of reduction in cue-induced freezing during extinction also did not differ between males and females. When relapse of extinguished fear was examined, however, male rats did not show renewal, reinstatement, and spontaneous recovery (Park et al., 2017b), which is consistent with previous findings suggesting that extinction is resistant to relapse in males at this age (Kim and Richardson, 2007a,b, 2010b; Gogolla et al., 2009), and may even erase the original fear memory (Kim and Richardson, 2008, 2009, 2010a; Gogolla et al., 2009). In contrast, females showed all three types of conditioned fear relapse following extinction (Park et al., 2017b). These findings suggest a resilient period early in life for males with effective extinction, while females may develop faster than males to be more vulnerable to persistent fear memory, which may explain human epidemiology (Kim, 2017).

Previous studies generally demonstrate that the increased involvement of the amygdala but reduced involvement of the prefrontal cortex (PFC) and the hippocampus explain the lack of relapse following extinction in juvenile male rodents compared to older rodents (Gogolla et al., 2009; Kim et al., 2009; Orsini et al., 2011; Ganella et al., 2018b; Li et al., 2018). Of these regions, the hippocampus in particular may explain the sex differences in relapse of extinguished fear observed in juvenile rats (Park et al., 2017a). Renewal, reinstatement, and spontaneous recovery are widely conceptualized as context-based relapse in which fear memory returns due to the physical, internal, and temporal context change from how extinction occurred (Bouton et al., 2006). Hippocampus is a well-studied region important for context learning (Kim and Fanselow, 1992; Lee et al., 2023), and its dysfunction during extinction can cause renewal (Corcoran et al., 2005). Hippocampus also changes rapidly during development and show sex-specific effects from infancy (Koss and Frick, 2016; Griffiths et al., 2019). However, temporary and bilateral inactivation of the dorsal or ventral hippocampus using an infusion of γ-aminobutyric acid agonist muscimol during extinction did not have sex-specific effects in P18 rats (Park et al., 2020b). In that study, dorsal hippocampus inactivation facilitated extinction acquisition in both sexes, while ventral hippocampus inactivation impaired extinction recall in both sexes. Renewal in females and the absence of renewal in males were not affected (Park et al., 2020b). Future studies assessing the amygdala and the PFC are necessary to understand the mechanisms driving the sex-specific effects in extinction in juvenile rodents.

## Adolescent sex differences in extinction of conditioned fear

Observation of impaired extinction recall in adolescent compared to preadolescent and adult rodents and humans (McCallum et al., 2010; Kim et al., 2011; Pattwell et al., 2012; Ganella et al., 2017a, 2018a) was a significant breakthrough that reflected the epidemiological and clinical characteristics in humans that report the highest onset of anxiety disorders and treatment resistance in adolescence (Kessler et al., 2007; Hartley and Casey, 2013). However, the original findings were exclusively in male rodents and sex-specific studies in adolescence are surprisingly scarce. Adolescence is a period of maturation marked by puberty and the onset of menarche in which estrous cycling is irregular (Perry et al., 2020). In the rodent brain, pubertal onset in female rats is marked by significant changes in cell and synapse numbers in the medial PFC at ~P35 while in males those changes are marked at ~P45 or more gradual across adolescence (Juraska and Willing, 2017; Drzewiecki and Juraska, 2020). In humans, female adolescents are arguably the most at-risk population to experience an anxiety disorder (Kessler et al., 2007; Craske et al., 2017). Consistent with such epidemiology, one study showed that estrous phase associated with the highest level of estradiol during extinction significantly impaired extinction in female adolescent rats (Perry et al., 2020). Specifically, females in proestrus or met/diestrus froze more than males during extinction, and females in met/diestrus froze more than males at extinction recall. Females in estrus or females that had not yet undergone menarche at extinction did not differ from males (Perry et al., 2020). Sex and estrus phase on conditioning day generally had no effects during acquisition and extinction of conditioned fear. Estrus phase on extinction recall day also had no effects on freezing levels on any behavioral day. Gonadectomy prior to the onset of puberty (i.e., at P21) facilitated extinction and improved extinction recall for female adolescent rats. In contrast to the females, gonadectomy produced delayed extinction in males, although extinction recall was unaffected. Similarly, a mouse study showed delayed extinction in adolescent females relative to males, with no sex differences reported for spontaneous recovery and renewal of extinguished fear (Lawson et al., 2022). In adolescence, there is a significant cortical reorganization related to sex hormones in rats that are more dramatic in females compared to males (Juraska and Drzewiecki, 2020), which may underlie females' sensitivity to estrous cycling and sex hormones in fear extinction. Overall, estrogen is detrimental while testosterone enhances extinction in adolescent rats, which may explain and model human epidemiology. This adolescent finding is contradictory to research in adults that estrogen is helpful for extinction (Milad et al., 2009), demonstrating the importance of age in understanding sex effects in extinction.

Interestingly, clear sex differences in extinction emerge in response to lifestyle factors in adolescence (Drummond et al., 2021). In that study, male adolescent rats reared in isolation showed impaired extinction recall, which was rescued by exercise during isolation. In adolescent females, isolation transiently disrupted conditioned fear acquisition, and exercise in isolation impaired extinction recall. These sex differences in response to isolation and/or exercise were unrelated to estrous cycling. Notably, neurogenesis in the ventral hippocampus positively correlated with extinction recall freezing levels in adolescent females but not in males, with chronic suppression of neurogenesis abolishing exercise effects in both sexes (Drummond et al., 2021). However, 4 days of alcohol drinking following extinction did not sex-specifically affect spontaneous recovery and renewal of extinguished fear (Lawson et al., 2022). Previous studies have shown that the balance between dopamine receptor 1 and 2 (D1 to D2) may be unique in adolescent males and females compared to other ages (Cullity et al., 2019; Bjerke et al., 2022), with extinction increasing D2 expression in adolescence and D1 expression in adulthood in PFC of male rats (Zbukvic and Kim, 2018). These findings strongly suggest hippocampus and PFC as potential mechanistic regions to explain sex differences in extinction in adolescence that should further be explored.

## Aging sex differences in extinction of conditioned fear

The PFC is a critical region for age-specific extinction (Ganella et al., 2017b; Kim et al., 2017; Zbukvic et al., 2017; Zbukvic and Kim, 2018; Perry et al., 2021). In humans, the PFC is one of the first to atrophy in aging, which is consistently related to reduced cognitive flexibility (Armstrong et al., 2019; Cui et al., 2023). Extinction could be considered a test of cognitive flexibility because it involves the flexible retrieval of the fear memory or the extinction memory depending on the appropriate circumstance (Kaczorowski et al., 2012; Short et al., 2022). Sex differences in aging PFC have been reported in longitudinal studies, with males showing greater age-related thinning than females when observed over 10 years (Pacheco et al., 2015), although other studies with shorter observational periods have reported no sex differences (Yuan et al., 2018). These findings suggest that extinction may sex-specifically change during aging, however, the studies are scarce in any species.

In rats, extinction recall was impaired in middle-aged (13–18 months old) and aged (22–28 months old) compared to adults (3–6 months old), which was associated with decreased excitability in regular spiking neurons in the infralimbic cortex of PFC and increased excitability in burst spiking neurons in the prelimbic cortex of PFC (Kaczorowski et al., 2012). These findings are highly insightful because the infralimbic cortex is generally considered to drive fear reduction whereas the prelimbic cortex is considered to drive fear expression (Maren et al., 2013). However, that study only examined males, and it is only recently that both sexes were tested for extinction in aging rodents. Specifically, aging from adulthood (3 months old) to middle-age (11 months old) increased conditioned fear expression similarly across male and female mice (Short et al., 2022), which is consistent with the increased excitability in the prelimbic cortex reported previously (Kaczorowski et al., 2012). Conditioned freezing remained higher in aged mice during fear retrieval and extinction recall (Short et al., 2022). Further, middle-aged males showed higher levels of conditioned fear retrieval but more rapid extinction acquisition compared to females. Adult mice showed robust reinstatement of extinguished fear, whereas middled-aged mice did not show any reinstatement. There were no sex effects in reinstatement, however, access to running wheels from 8 months of age rescued reinstatement in male but not female middle-aged mice. In that study, hippocampal brain-derived neurotrophic factor (*Bdnf*) mRNA levels were measured after reinstatement test. Increased hippocampal *Bdnf* expression in freely exercising rodents is a well-established molecular correlate for neuronal growth and survival relevant for exercise-associated benefits on brain and behavior (Neeper et al., 1996; Berchtold et al., 2001; Cotman et al., 2007). Surprisingly, aging did not affect hippocampal brain-derived neurotrophic factor (Bdnf) mRNA levels, although mice with running wheel access showed increased total Bdnf and Bdnf exon 4 mRNA levels in both sexes (Short et al., 2022).

Taken together, sex effects were only observed during extinction acquisition or following exercise in aging mice (Short et al., 2022). Therefore, continued exploration of the PFC and the hippocampus processes, as well as other regions important for extinction acquisition, such as the amygdala (Li et al., 2009; Madsen et al., 2017), are required to understand the mechanisms underlying sex effects in aging. In addition, 11 months of age in rodents are considered peri-menopausal and peri-andropausal (Lu et al., 1979), in which hormone levels fluctuate not unlike adolescence. Sex hormones assessments of extinction in aging rodents is critical to understand the biology underlying fear and anxiety in aging, which may contribute to the increased duration of anxiety disorders and reduced function in aging human males (Preville et al., 2010; Vasiliadis et al., 2020).

## Conclusions

Age- and sex-specific preclinical research in extinction is clearly relevant for effective research translation, with many observations more clearly corresponding to clinical and epidemiological characteristics of anxiety disorders than sex effects reported in adults. For example, juvenile female rodents show relapse of extinguished fear when males do not and high levels of estrogen in adolescent female rodents are detrimental to extinction. These findings can explain the higher prevalence of anxiety disorders observed in females over males. We recommend researchers not to only include both sexes but also to analyze and report statistical findings concerning sex, considering that many studies now include both sexes but often sex-specific and sex difference analyses are unreported. The dearth of studies that report sex-specific findings in extinction, with no information on critical periods such as before and after menopause/andropause, is a glaring research gap that we should strive to address.

## Author contributions

SM: Visualization, Writing – original draft. CP: Visualization, Writing – review & editing. JK: Conceptualization, Funding acquisition, Supervision, Visualization, Writing – original draft, Writing – review & editing.
